# Analgesic outcomes of tramadol alone and in combination with Butorphanol or Flurbiprofen Axetil after cesarean section: a retrospective study with propensity score matching analysis

**DOI:** 10.1186/s12871-022-01939-4

**Published:** 2022-12-16

**Authors:** Guiying Yang, Zhuoxi Wu, Qiangting Deng, Yan Liang, Xiaohang Bao, Guangming Yan, Jing Peng, Wenjun Liu, Dan Tan, Hong Li

**Affiliations:** 1 Department of Anesthesiology, Second Affiliated Hospital of Army Medical University, Chongqing, 400037 China; 2 Editorial Office of Journal of Army Medical University, Chongqing, 400038 China; 3 Department of Emergency, Second Affiliated Hospital of Army Medical University, Chongqing, 400037 China

**Keywords:** Cesarean section, Analgesia, Tramadol, Flurbiprofen axetil, Butorphanol

## Abstract

**Background:**

The Society for Obstetric Anesthesia and Perinatology recommends a multimodal analgesia regimen for cesarean delivery analgesia. This study aimed to compare the analgesic effects of tramadol alone and combined with butorphanol or flurbiprofen axetil after a cesarean section.

**Methods:**

We performed a retrospective analysis based on the electronic medical records of a teaching hospital in China from January 2018 to January 2020. We collected data on demographic characteristics, anesthesia, analgesia strategy, and pain intensity postoperatively during the first 48 hours. Inadequate postoperative analgesia during this period was defined as an NRS score ≥ 4. We also collected data regarding off-bed activity and intestinal function recovery. Participants were classified into three groups according to analgesia regimens. Groups T, TF, and TB received tramadol, a mixture of tramadol and flurbiprofen axetil, and a combination of tramadol and butorphanol, respectively. Analgesic outcomes were compared using propensity score matching analysis.

**Results:**

Data from 2323 cases of caesarean section were included in the analysis, and 521 pairs were matched in each group according to their propensity score. Compared with group T, The inadequate analgesia on pain at rest and pain at movement was lower in group TF (RR: 0.42, 95% CI: 0.36–0.49, *P* = 0.001 and RR: 0.58, 95% CI: 0.48–0.69, *P* < 0.001, respectively),and the incidence of inadequate control of pain at movement was higher in group TB (RR: 1.38, 95% CI: 1.22–1.55, *P* < 0.001).

Additionally, the percentage of off-bed activity at 2 days postoperatively was higher in group TB than in groups TF and T (78.7% vs. 68.5 and 78.7% vs. 64.9%, respectively, *P* < 0.001). The incidence of intestinal function recovery 2 days after cesarean delivery in group TB was higher than that in group TF (73.3% vs. 66.2%, *P* = 0.013).

**Conclusions:**

Combining tramadol and flurbiprofen axetil could enhance the analgesic effect and be safely used for analgesia after a cesarean section. However, combining tramadol and butorphanol may produce an antagonistic effect.

## Background

Cesarean section is the most common surgery globally. Inadequate analgesia after cesarean section is common [1, 2], and the average numeric rating scale (NRS) score is > 6 on day 1 postoperatively [3]. Therefore, researching the analgesic strategy after a cesarean section is very important. Neuraxial analgesia provides optimal pain relief after a cesarean section through patient-controlled epidural analgesia (PCEA) [4]. However, epidural catheter dislocation [5] or slippage [6] and the infection risk [7] limit the clinical application of PCEA. Patient-controlled intravenous analgesia (PCIA) offers another pain control method after a cesarean section without these complications. However, in the Practice Bulletin of Obstetric Analgesia and Anaesthesia 2019, no optimal intravenous analgesia strategy was recommended for pain treatment in females who have undergone a cesarean section [8].

Tramadol is a usually used analgesic in China after a cesarean section [9, 10]. Females who undergo cesarean section are at high risk for postpartum depression. Our preliminary studies suggest that PCIA with tramadol could alleviate anxiety and depression in the early postpartum period [10, 11]. Thus, it is used as the main analgesic drug administered after a cesarean section. However, tramadol has no obvious analgesic effect on visceral pain.

Females who have had a cesarean section have both incision and visceral pain. Oxytocin is routinely used in clinical practice to reduce uterine bleeding after a cesarean section, and uterine cramping pain (UCP) is induced simultaneously. Butorphanol is both a kappa-agonist and a mu-antagonist analgesic and is associated with less respiratory depression in an equianalgesic dose. Hence, butorphanol provides optimal visceral pain relief after cesarean sections. In addition, it is safe for breastfeeding females in the early postpartum period; hence, it is widely used for analgesia after cesarean section [12, 13]. Therefore, we thought that combining tramadol with other analgesic drugs that have an analgesic effect on visceral pain may be a good choice for females with cesarean section.

Current studies have suggested that non-steroidal drugs positively affect UCP [14]. For example, flurbiprofen axetil is a non-selective non-steroidal drug, and it is safe for females who are breastfeeding in the early postpartum period [15, 16]; thus, it is also safe for cesarean section analgesia.

To our knowledge, the Society for Obstetric Anesthesia and Perinatology recommends multimodal analgesia (MMA) regimen for cesarean delivery analgesia. Moreover, it is beneficial for patients to choose a combination of analgesics that is effective and safe for both the mother and newborn. Therefore, we conducted a retrospective study on the postoperative analgesic effects of tramadol alone and in combination with flurbiprofen axetil or butorphanol after cesarean section and conducted propensity score matching (PSM) analysis to explore the differences in postoperative analgesia effects among the drug combinations.

## Methods

### Study design and patients

This single-center retrospective cohort study investigated the postoperative analgesia effects of tramadol alone and in combination with flurbiprofen axetil or butorphanol after cesarean section. The study protocol was approved by the Institutional Ethics Committee of the Second Affiliated Hospital of Army Medical University, Chongqing, China (approval ID: 2020–101-01). Written informed consent was not required from all the participants.

**Fig. 1:**
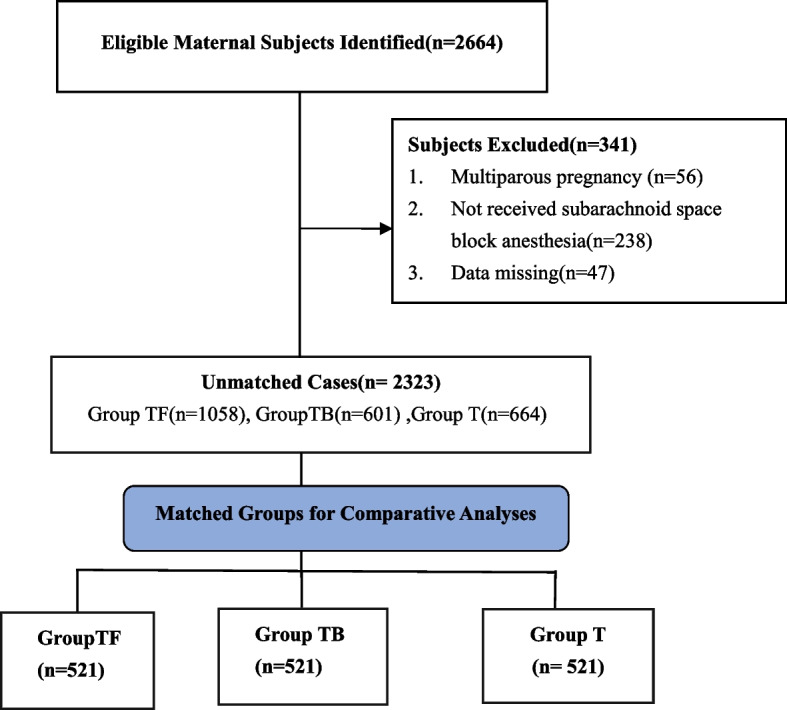
Flow diagram of patient inclusion

The inclusion criteria were cases of caesarean section with a gestational age of 37–40 weeks, an American Society of Anesthesiology physical status of I–II, and singleton pregnancy. The exclusion criteria included multiple pregnancies and missing data (if one missing data on one variable means exclusion). As shown in Fig. 1, from January 2018 to January 2020, we enrolled a total of 2323 Chinese cases of caesarean section aged 20–44 years who had undergone nonemergency cesarean section under subarachnoid space block anesthesia.

### Patient data and outcomes collection

#### Data source

All data were based on the electronic medical records of The Affiliated Hospital of Army Medical University from January 2018 to January 2020. A gynecologist recorded baseline data and preoperative demographics. An anesthetist recorded anesthesia-related data and intraoperative complications, and a nurse anesthetist performed postoperative follow-up.

#### Analgesia and anesthesia

An experienced anesthesiologist administered anesthesia. Spinal anesthesia was used for all the patients, and ropivacaine (AstraZeneca AB; 18–22 mg) was administered at the puncture site (L2–L3 or L3–L4 level). Postoperative analgesia was started immediately after the cesarean section, and PCIA was performed through a mechanical infusion pump. Three analgesia strategies were selected. The selection of different analgesic strategies is determined by the drug inventory (the supply of drugs varies at different time period), and patients were classified into three groups according to three analgesic regimens. The tramadol group (group T) received a mixture of tramadol (Sandoz [China] Pharmaceutical Co., Ltd.; 800 mg) with 0.9% normal saline at a dose volume of 200 mL, flurbiprofen axetil group (group TF) received a mixture of tramadol (800 mg) and flurbiprofen axetil (Teide Pharmaceutical Co., Ltd., Beijing, China; 200 mg) with 0.9% normal saline at a dose volume of 200 mL, and butorphanol group (group TB) received a combination of tramadol (800 mg) and butorphanol (Hengrui Pharmaceutical Co., Ltd., Jiangsu, China; 2 mg) with 0.9% normal saline at a dose volume of 200 mL for PCIA. The PCIA pump was designed to have a background infusion rate of 4.0 mL/hour and an additional dose of 1 mL with a lockout period of 15 minutes.

#### Outcomes

Postoperative pain intensity was evaluated using the NRS (score: 0–10; with 0 as no pain and 10 as maximum pain) at 6, 12, 24, and 48 hours. The primary outcome was the incidence of inadequate postoperative analgesia on pain at movement during the first 48 hours after a cesarean section because it is considered an important basis for using supplementary analgesia in clinical practice. Inadequate analgesia was defined as an NRS score ≥ 4 during the first 48 hours postoperatively. In addition, the number of patients who requested extra rescue pain treatment during the postoperative analgesia was recorded.

We also recorded the following maternal preoperative complications for all the included cases of caesarean section:

- Gestational diabetes mellitus, including previously diagnosed diabetes and emerging diabetes during the gestational period;
- Gestational hypertension, which included pre-pregnancy hypertension, emerging hypertension during pregnancy, pre-eclampsia, or eclampsia;
- Oligoamnios and polyhydramnios, which are defined as amniotic fluid volumes < 300 mL and > 2000 mL, respectively, during pregnancy;
- Placental diseases, including placenta previa and placenta abruption;
- Fetal macrosomia, which is defined as a fetus weighing ≥4000 g;
- Pre-diagnosed fetal diseases, including fetal congenital brain disease, heart disease, and kidney disease, such as hydrocephalus and hydronephrosis, etc.;
- Premature membrane rupture, which refers to membrane rupture before regular uterine contractions;
- Synchronized surgery, for cases of caesarean section who underwent other surgeries during the cesarean section, such as simultaneous ligation, ovarian cyst surgery, uterine fibroid surgery, etc.

Demographic data such as age and body mass index (BMI), as well as data on intraoperative blood loss volumes, were collected. Additionally, we recorded patients in whom intrauterine balloon tamponade was used for uterine inertia and postpartum hemorrhage treatment. Moreover, postoperative off-bed activity and intestinal function recovery 2 days after surgery, length of hospitalization stay, and the hospitalization cost were recorded.

### Statistical analysis

All data were analyzed using SPSS 26.0 (IBM Corp.) and R software (version 3.0.1; http://www.Rproject.org). A two-sided *P*-value < 0.05 was considered to be statistically significant. Continuous variables are presented as mean ± standard deviation (SD), while number (frequency) is used to summarize categorical variables.

A stepwise logistic regression analysis was used to evaluate the role of demographic and preoperative baseline data variables in predicting inadequate postoperative analgesia. Furthermore, all demographic and preoperative baseline data variables in Table 1 were included in this model. Odds ratio (OR) with a 95% confidence interval (CI) were ascertained based on the logistic regression analysis.

**Table 1:** Demographic and preoperative baseline data for three groups

	non-matched patients				matched patients				
	Group TF(*n* = 1058)	Group TB(*n* = 601)	Group T (*n* = 664)	*P* values	Group TF(*n* = 521)	Group TB (*n* = 521)	Group T (*n* = 521)	*P* values	SMD
Age (year)	30.4 ± 4.4	30.4 ± 6.2	30.6 ± 4.6	0.423	30.4 ± 4.4	30.1 ± 4.4	30.1 ± 4.3	0.541	0.065
BMI (kg/m^2^)	27.7 ± 3.9	27.6 ± 3.4	27.7 ± 3.4	0.831	27.4 ± 3.4	27.6 ± 3.4	27.6 ± 3.4	0.520	0.063
Duration of operation (min)	84 ± 34	80 ± 25	79 ± 23	0.002	81 ± 26	80 ± 25	80 ± 23	0.356	0.085
Intraoperative blood loss (mL)	341 ± 268	308 ± 188	325 ± 207	0.020	207 ± 9	195 ± 8	195 + 4	0.376	0.085
Placenta disease	89 (8.4%)	34 (5.7%)	66 (9.9%)	0.015	52 (10.0%)	33 (6.3%)	35 (6.7)	0.052	0.157
Oligoamnios or polyhydramnios	72 (6.8%)	34 (5.7%)	31 (4.7%)	0.179	21 (4.0%)	25 (4.8%)	30 (5.8)	0.430	0.080
Prediagnosed fetal disease	17 (1.6%)	14 (2.3%)	11 (1.7%)	0.536	9 (1.7%)	6 (1.2%)	11 (2.1%)	0.485	0.076
Premature rupture of membrane	101 (9.6%)	76 (12.7%)	69 (10.4%)	0.140	45 (8.6%)	57 (10.9%)	62 (11.9%)	0.210	0.108
Hypertension	80 (7.6%)	39 (7.0%)	40 (6.0%)	0.433	21 (4.0%)	33 (6.3%)	29 (5.6%)	0.240	0.104
Gestational diabetes	108 (10.2%)	71 (11.8%)	70 (10.6%)	0.588	62 (11.9%)	54 (10.3%)	56 (10.75)	0.712	0.049
Hyperthyroidism or hypothyroidism	62 (5.9%)	39 (6.5%)	39 (5.9%)	0.858	33 (6.3%)	32 (6.1%)	30 (5.8)	0.925	0.024
History of surgery	19 (1.8%)	9 (1.5%)	12 (1.8%)	0.886	13 (2.5%)	8 (1.5%)	9 (1.7%)	0.490	0.068
Intrauterine Balloon Tamponade	120 (11.3%)	57 (9.5%)	54 (8.1%)	0.087	63 (12.1%)	46 (8.8%)	41 (7.9%)	0.053	0.141
Synchronized other surgery	115 (10.9%)	61 (10.5%)	69 (10.4%)	0.990	59 (11.3%)	53 (10.2%)	49 (9.4%)	0.591	0.063
Primiparas or multiparas	477 (45.1%)	247 (41.1%)	251 (37.8%)	0.010	218 (41.8%)	211 (40.5%)	212 (40.7%)	0.893	0.027

Data were presented as Means ± SD or as numbers (frequency); *BMI* body mass index, *SMD* standardized mean difference

a: *P*-value obtained via comparison of three groups

b: ANOVA was used to compare normally distributed data, and chi-square test or Fisher exact were used to compare proportions

All groups were matched by propensity scores to avoid the influence of bias and confounding variables. Additionally,becausen all demographic and preoperative baseline data variables in Table 1 might be important factors to affect postoperative pain scores, these variables were used to calculated the propensity scores. The propensity score was calculated for the following baseline variables: age, BMI, duration of operation, placenta disease (yes/no), intraoperative blood loss, gestational diabetes (yes/no), other synchronized surgery (yes/no), primiparas or multiparas, oligoamnios or polyhydramnios, pre-diagnosed fetal disease (yes/no), premature rupture of membrane (yes/no), hypertension (yes/no), hyperthyroidism or hypothyroidism (yes/no), history of surgery (yes/no), and intrauterine balloon tamponade (yes/no). The packages ‘foreign,’ ‘dplyr,’ ‘RMS,’ and ‘VIM’ in R language (version 3.0.1; http://www.Rproject.org) were used to perform propensity score matching (PSM) between three groups for statistical analysis. Matching was done using the 1:1:1 nearest method, and 0.2 was set as the caliper value. We conducted propensity score matching (PSM) according to the method used by Deng Qiangting et al. [17].

Normally distributed continuous data among three groups were compared using ANOVA, including age, BMI, duration of operation, intraoperative blood loss, hospitalization stay, and all hospitalization and pain treatment costs. Furthermore, the Tukey post hoc analysis was used for continuous variables comparison between each matched group, including hospitalization stay, and all hospitalization and pain treatment costs. Categorical variables among three groups were compared using the chi-square or Fisher exact tests. The chi-square or Fisher exact tests were also used again for comparison between each matched group. Additionally, and a two-sided *P*-value < 0.01666 was significance threshold for each of the matched group comparison of categorical variables. Furthermore, the relative risks (RRs) values for the occurrence of inadequate analgesia during the first 48 hours postoperatively were calculated with a 95% CI.

## Results

### Demographic and preoperative baseline data analysis

Two thousand three hundred twenty-three patients were identified in this study: 1058 in group TF, 601 in group TB, and 664 in group T. The demographic and preoperative baseline data of the three groups are shown in Table 1. There were significant between-group differences in duration of operation, blood loss during the operation, the incidence of placenta disease, and primiparas or multiparas (all, *P* < 0.05). However, no significant difference was observed in other preoperative parameters (*P* > 0.05). After PSM, 521 patients were matched in each group, and no significant difference in the demographic and preoperative baseline data was observed among groups (*P* > 0.05).

### Risk factor analysis

As summarized in Table 2, for all included cases of caesarean section, the results of stepwise logistic regression analysis showed that placenta disease was risk factor of inadequate analgesia for pain while at movement (OR: 2.30, 95% CI: 1.51–3.50, *P* < 0.001). Intraoperative blood loss was identified as significant factors for inadequate analgesia for pain at movement (OR: 1.00, 95% CI: 1.00–1.01, *P* < 0.001).

**Table 2:** Factors associated with inadequate analgesia in Stepwise Logistic Regression Analysis for all groups

Subjects	Outcome	Factors with statistical significance	Wald	*P* value	OR	95% CI
All maternal women (*n* = 2323)	Inadequate analgesia on pain at movement	Placenta disease	15.08	< 0.001	2.30	1.51 to 3.50
		Intraoperative blood loss	15.22	< 0.001	1.00	1.00 to 1.01

*CI* Confidence interval, *OR* Odds rate

a: All demographic and preoperative baseline data variables was used as dependent variable and inadequate analgesia was used as outcome variable

b: *P* values obtained via a stepwise logistic regression analysis

### Outcomes analysis among the three groups

As shown in Table 3, among the three groups, significant differences were observed in the incidences of inadequate analgesia (pain at rest and pain at movement), additional analgesia requirement, off-bed activity, and intestinal function recovery 2 days after cesarean delivery (*P* < 0.05). A significant among-group difference was also observed in hospitalization stay and total hospitalization and pain treatment costs (yuan) (*P* < 0.05). However, there were no significant differences in dizziness, nausea, or vomiting (*P* > 0.05).

**Table 3:** Intraoperative and postoperative outcomes for matched patients among three groups

	Group TF(*n* = 521)	Group TB (*n* = 521)	Group T (*n* = 521)	*P* values among three groups	*P* values for each mached group		
					TF vs. TB	TF vs. T	TB vs. T
Inadequate analgesia (pain at rest)	25 (4.8%)	65 (12.5%)	54 (10.4%)	< 0.001	< 0.001	0.001	0.284
Inadequate analgesia (pain at movement)	129 (24.8%)	308 (59.1%)	224 (43.0%)	< 0.001	< 0.001	< 0.001	< 0.001
Extra analgesia requirement	5 (1.0%)	15 (2.9%)	5 (1.0%)	0.017	0.024	1.000	0.024
Off-bed activity at 2 day after cesarean delivery	357 (68.5%)	410 (78.7%)	338 (64.9%)	< 0.001	< 0.001	0.212	< 0.001
Intestinal function recovery at 2 day after cesarean delivery	345 (66.2%)	382 (73.3%)	363 (69.7%)	0.044	0.013	0.232	0.666
Hospitalization stay (day)	3.9 ± 1.4	4.3 ± 1.6	4.2 ± 1.7	< 0.001	< 0.001	< 0.001	0.845
All Hospitalization cost (yuan)	13,669 ± 4244	13,389 ± 4624	15,276 ± 4420	< 0.001	0.693	< 0.001	< 0.001
Pain treatment cost (yuan)	160 ± 1	158 ± 2	86 ± 1	< 0.001	< 0.001	< 0.001	< 0.001

Data were presented as Means ± SD or as numbers (frequency)

a: *P*-value obtained via comparison of each matched group, *P* < 0.05 and *P* < 0.01666 were significance threshold for continuous variables and categorical variables, respectively

b: ANOVA was used to compare continuous variables among three groups, post hoc analysis was used to compare continuous variables,and chi-square test or Fisher exact were used to compare proportions

### Comparison based on the outcomes of each matched group

As shown in Table 3, compared with group T, the inadequate analgesia on pain at rest and pain at movement was lower in group TF (RR: 0.42, 95% CI: 0.36–0.49, *P* = 0.001 and RR: 0.58, 95% CI: 0.48–0.69, *P* < 0.001, respectively), and the incidence of inadequate control of pain at movement was higher in group TB (RR: 1.38, 95% CI: 1.22–1.55, *P* < 0.001).

The percentage of off-bed activity at 2 days postoperative was higher in group TB than that in groups TF and T (78.7% vs. 68.5 and 78.7% vs. 64.9%, respectively, *P* < 0.001). In addition, the incidence of intestinal function recovery 2 days after cesarean section in group TB was higher than that in group TF (73.3% vs. 66.2%, *P* = 0.013).

The length of hospitalization stay was lower in group TF than in groups TB and T (*P* < 0.001). In addition, pain treatment cost in group T was lower than in groups TF and TB (*P* < 0.001), but the total hospitalization cost was higher than that in the other two groups (*P* < 0.001).

## Discussion

To the best of our knowledge, this is the first study to explore the potential differences in postoperative analgesia among tramadol alone and tramadol combined with butorphanol or flurbiprofen axetil after cesarean section through retrospective analysis. Our results were confirmed in the propensity score-matched cohort after using PSM analysis. This finding shows that tramadol combined with flurbiprofen axetil had an optimal analgesic effect, and the incidence of inadequate analgesia was highest with tramadol combined with butorphanol. However, in group TB, off-bed activity and intestinal function recovery after cesarean section occurred the earliest. However, it should be noted that continuous background dose of PCIA was used in this study, although this is banned in some country, but it is commonly used in China [9–11], and drug overdoses were not discovered in our clinical practice.

MMA was advocated for postoperative analgesia after cesarean section, including combinations of analgesic medications that act on different pathways and sites synergistically or additively to relieve pain with minimal or no opioid consumption. Since butorphanol, flurbiprofen axetil and tramadol are medications with different pathways of action, the doses of the three tramadol suites were all 800 mg without reduction.

The combination of low-dose opioids and non-opioids was a method for postoperative analgesia in patients with large surgical incisions. Cesarean section involve surgery with a large incision and intense postoperative pain. Therefore, it is beneficial to choose a combination of opioids and non-opioids that is both effective and safe for mothers and newborns. However, there are many options for analgesic medication combination strategies, and it is uncertain which of these strategies is optimal. Tramadol combined with flurbiprofen axetil is one of the analgesia strategies for cesarean section. Our findings revealed that tramadol combined with flurbiprofen axetil has the optimal analgesic effect for patients with cesarean sections. Tramadol is an effective analgesic for cesarean section and has been shown to reduce the incidence of postpartum depression [10, 11]. Furthermore, flurbiprofen axetil plays analgesic and anti-inflammatory effects by reducing the release of prostaglandins associated with UCP [18, 19]. Hence, co-administration of tramadol and flurbiprofen axetil has a definite analgesic effect for cesarean section [20, 21]. This study also found the pain treatment cost in group TF was higher than that in the other two groups. Nonetheless, there was no increase in total hospital cost, which indicated that group TF did not increase the financial burden on patients. In conclusion, the analgesia strategy of tramadol combined with flurbiprofen axetil is a good choice for obstetric analgesia. However, it should be noted that NSAIDs is effective both on visceral pain [22] and on pain at movement [23] after cesarean section, but strangely not converted enough into current practice, and breastfeeding is often claimed as a limitation of use in post-caesarean. There are data supporting such practice [24, 25], so low doses can be used if they are added to paracetamol [26, 27].

The United States Food and Drug Administration recommends avoiding the co-administration of mixed agonist-antagonists and full opioid agonist use because of the diminishing analgesic effect. One study also reported that co-administration of tramadol with butorphanol did not provide additional analgesic effects [28]. Another animal study reported that the side effects of hydromorphone could be reduced by butorphanol and the degree of reduction is dose-dependent [29]. Nonetheless, some anesthesiologists prefer to co-administer agonist-antagonists and agonists for postoperative analgesia [30, 31]. They believe this method can enhance the analgesic effect while reducing the adverse reactions of opioids for postoperative analgesia.

Tramadol is a mu receptor agonist, and butorphanol is an agonist-antagonist analgesic drug. Some anesthesiologists use tramadol combined with butorphanol for postoperative analgesia or as analgesic supplementation [32, 33]. One study suggests that tramadol combined with butorphanol had a stronger analgesic effect for UCP than sufentanil for cesarean section analgesia in an equivalent dose; however, no significant difference was detected for incision pain [34]. Hence, no significant antagonistic effect was found in their study. Therefore, it is necessary to further explore the analgesic effect of the combination of these two drugs. Our results indicate that the incidence of inadequate analgesia was higher in group TB (800 mg of tramadol combined with 2 mg of butorphanol for PCIA in 2 days) than in groups TF and T, but off-bed activity and intestinal function recovery occurred earliest in group TB. We hypothesized that butorphanol produced an antagonistic effect on tramadol, which attenuated the analgesic effect and reduced the adverse effects of inhibiting gastrointestinal function. Therefore, whether the analgesic method of butorphanol combined with tramadol is reasonable and whether we should change the analgesic strategy is worth further consideration.

Several limitations should be considered when evaluating the results of the present study. First, some limitations in data collection were unavoidable, given the study’s retrospective nature. Second, since it is difficult to distinguish between visceral pain and incision pain, visceral pain was not recorded separately, so this should be further studied in the future. Third, Since this is a retrospective study, there were no data on patients using butorphanol combined with flurbiprofen axetil, which needs to be discussed in subsequent studies. Fourth,a mechanical pump was used for PCIA in our hospital. Because the pump could not record the analgesic consumption, we did not report the analgesic use between the groups in this study; we only recorded the additional analgesia requirement. Finally, Opioids were not used in spinal anesthesia in this study, so this study lacks generalizability to postpartum populations who received intrathecal opioids for pain control.

## Conclusion

In conclusion, a combination of tramadol and flurbiprofen axetil enhanced the analgesic effect and was safely used for analgesia after cesarean section. However, the combination of tramadol and butorphanol may produce an antagonistic effect.

## Data Availability

All data can be acquired from the corresponding author (HL) by request.

## Abbreviations

OR: Odds ratio
RRs: relative risks
CI: Confidence interval
BMI: body mass index
PCIA: patient-controlled intravenous analgesia
PCEA: patient-controlled epidural analgesia
OT: oxytocin
CP: uterine cramping pain
MMA: multimodal analgesia
SMD: standardized mean difference
